# GLUT5: structure, functions, diseases and potential applications

**DOI:** 10.3724/abbs.2023158

**Published:** 2023-09-07

**Authors:** Aqian Song, Yuanpeng Mao, Hongshan Wei

**Affiliations:** 1 Department of Gastroenterology Beijing Ditan Hospital Capital Medical University Beijing 100015 China; 2 Department of Gastroenterology Peking University Ditan Teaching Hospital Beijing 100015 China

**Keywords:** GLUT5, fructose, intestinal disease, cancer

## Abstract

Glucose transporter 5 (GLUT5) is a membrane transporter that specifically transports fructose and plays a key role in dietary fructose uptake and metabolism. In recent years, a high fructose diet has occupied an important position in the daily intake of human beings, resulting in a significant increase in the incidence of obesity and metabolic diseases worldwide. Over the past few decades, GLUT5 has been well understood to play a significant role in the pathogenesis of human digestive diseases. Recently, the role of GLUT5 in human cancer has received widespread attention, and a large number of studies have focused on exploring the effects of changes in GLUT5 expression levels on cancer cell survival, metabolism and metastasis. However, due to various difficulties and shortcomings, the molecular structure and mechanism of GLUT5 have not been fully elucidated, which to some extent prevents us from revealing the relationship between GLUT5 expression and cell carcinogenesis at the protein molecular level. In this review, we summarize the current understanding of the structure and function of mammalian GLUT5 and its relationship to intestinal diseases and cancer and suggest that GLUT5 may be an important target for cancer therapy.

## Introduction

In the last few decades, high fructose corn syrup (HFS), which comprises high concentrations of fructose, has been widely used around the world due to developments and expansions in sugar production processes [1]. This has led to a dramatic increase in the global per capita intake of fructose, which has become a dominant component of the human diet and correlates closely with the increased incidence of cancers and metabolic diseases [ 2 – 4]. According to data published by the World Health Organization, 13% of adults worldwide are obese [5], which undoubtedly significantly increases the incidence of certain diseases. The proliferation of metabolic diseases and cancers caused by high fructose intake and obesity has created a huge economic and medical burden worldwide, and this serious public health problem has led to calls to limit fructose intake [6]. At the same time, this also means that the study of fructose metabolic pathways and their transport carriers GLUTs cannot be delayed [7]. 

Mammals express 14 GLUTs: GLUT1–14, all of which are members of the solute carrier 2A ( *SLC2A*) gene family [8]. Currently, all known GLUTs are divided into three classes ( Table 1). Class I comprises GLUT1-4 and GLUT14. Class II comprises GLUT5, 7, 9, and 11. Class III includes GLUT6, 8, 10, 12 and HMIT1 [ 9, 10]. In addition to GLUT13, other GLUTs mediate the facilitated diffusion (passive transport) of glucose or fructose [11]. Among the entire family of GLUTs, GLUT5 is the only transporter that specifically transports fructose, encoding the gene *SLC2A5* (chromosome localization 1p36.23), originally cloned from the human small intestinal cDNA library [12]. Human GLUT5 has a high affinity for fructose ( *K*_m_=6 mM) and no transport activity for glucose or galactose. Due to this characteristic, only GLUT5 is discussed in this paper.

**Table:** **Table 1** Classification, main expression, and main substrates of the 14 known GLUTs

Classification	GLUTs	Main expression tissues	Main substrates	Function
Class I	GLUT1	Erythrocytes, blood-tissue barriers	Glucose, 2-DG	Basal uptake
	GLUT2	Liver, pancreas, small intestine	Glucose, fructose, glucosamine	Glucose sensing
	GLUT3	Neurons	Glucose, 2-DG	Neuronal uptake
	GLUT4	Adipocytes, muscle, heart	Glucose, glucosamine	Insulin-responsive
	GLUT14	Testis	Unknown	Duplicon of GLUT3
Class II	GLUT5	Small intestine, testis, muscle, kidney, erythrocytes	Fructose	Fructose transport
	GLUT7	Testis, small intestine, prostate	Glucose	Unknown
	GLUT9	Liver, kidney	Urate	Urate homeostasis
	GLUT11	Pancreas, kidney, placenta, muscle	Fructose, glucose	Unknown
Class III	GLUT6	Brain, spleen, leukocytes	Glucose	Lysosomal transport
	GLUT8	Testis, neurons, adipocytes	Glucose, trehalose	Trehalose transport
	GLUT10	Liver, pancreas	2-DG	Unknown
	GLUT12	Heart, prostate	Glucose	Insulin-responsive
	HMIT1	Brain	Myo-inositol	Myo-inositol transport

Adapted from the article by Reckzeh *et al* . [9].

2-DG: 2-deoxy-D-glucose; HMIT1: proton myo-inositol cotransporter.

## GLUT5 Is Involved in Dietary Fructose Metabolism

**Figure 1:**
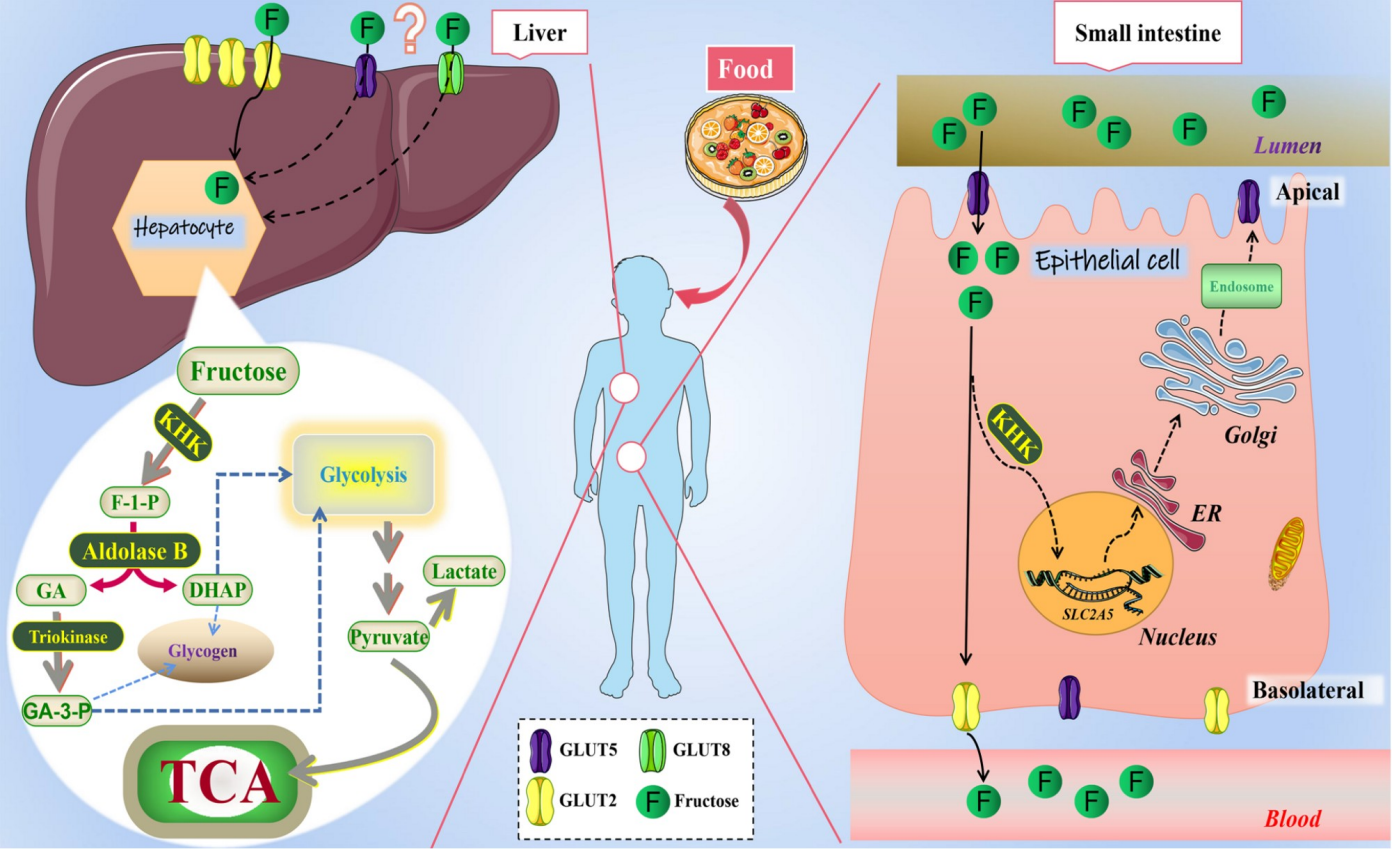
Figure 1 Metabolic process of fructose in small intestine and liver After the entry of fructose-rich foods into the human intestine, the fructose in the intestinal lumen, assisted by the GLUT5 transporter, enters the epithelium along the concentration gradient from the apical mo. Part of the fructose entering the cell is catabolized by KHK, and a series of products can activate SLC2A5 gene transcription in the nucleus. Most fructose is transported to the exocytosis by GLUT2 transporters on the basolateral membrane and enters the blood circulation. Fructose absorbed from the intestine reaches the liver through the portal vein. On the surface of hepatocytes, mainly GLUT2 transporters are responsible for transporting fructose into the cells, and possibly GLUT5 and GLUT8 are also involved in the transport process. The fructose that enters the hepatocytes is then phosphorylated to F-1-P by KHK. Aldolase B hydrolyzes F-1-P to GA and DHAP, and GA can be further phosphorylated to GA-3-P by triokinase. Both GA-3-P and DHAP can enter the glycolysis pathway directly to produce pyruvate. Pyruvate can be further metabolized to produce lactate or enter the mitochondrial TCA cycle. GA-3-P and DHAP can also synthesize glycogen through gluconeogenesis [13–17].

How does the fructose from food make its way from the gut into the bloodstream and end up in tissues and organs to be utilized? This involves the synergy of GLUT5, several other GLUTs, and several enzymes. Dietary fructose metabolism begins with absorption from the small intestine. Fructose in food will lead to an increase in the fructose concentration in the intestinal lumen, accordingly promoting fructose transmembrane transport and fluctuating around GLUT5 *K*_m_ [13]. After processing and maturation in the Golgi apparatus, the newly synthesized GLUT5 is transported to the apical and basolateral membranes of small intestinal epithelial cells with the assistance of circulating endosomes mediated by cytosolic Ras-related protein in brain 11a (Rab11a), a small GTPase that plays an essential role in the transportation of apical proteins in the intestine [ 14, 15]. GLUT5 is the carrier for facilitated diffusion of fructose into epithelial cells through the intestinal lumen, but GLUT2 is responsible for most fructose transport across the intestinal basal lateral membrane to the extracellular and circulates [16]. A small fraction of fructose is phosphorylated to fructose-1-phosphate (F-1-P) by ketohexokinase (KHK) in the cytoplasm, which is conducive to maintaining the fructose concentration gradient from the intestinal lumen to the cytosol, and the subsequent reaction products can stimulate *SLC2A5* transcription in the nucleus and GLUT5 mRNA translation and facilitate the continuous transport of fructose to the cell membrane [17] ( Figure 1). Two forms of the enzymes, KHK-a and KHK-c, are encoded by the *KHK* gene [18]. The affinity of KHK-c for fructose ( *K*_m_=0.8 mM) is 10-fold higher than that of KHK-a, and it is widely distributed in various tissues and organs of humans and is the most important enzyme responsible for fructose phosphorylation [ 19, 20]. Benign fructosuria is caused by mutations in the *KHK* gene. Individuals with this disorder will experience significant changes in circulating and urine fructose levels after consuming fructose-containing foods: after oral or intravenous injection of fructose, circulating fructose levels are consistently higher, much higher than levels in controls, and then slowly decline, with approximately 20 percent of fructose eventually excreted from the urine, compared with 1 to 2 percent in normal subjects [21].

Circulating fructose enters the liver via the portal vein, where most of the fructose is metabolized, which also keeps the serum fructose level at a low state all the time [22]. The first-pass metabolic role of the liver makes it the primary site of fructose metabolism in humans, but recent theories suggest that the importance of visceral organs in individual fructose metabolism is related to their organ size [23]. However, Jang and his colleagues [24] followed the metabolic process of fructose in mice by isotope tracing and came to a surprising conclusion: the small intestine could protect the liver from toxic fructose. However, it remains unclear whether intestinal fructose metabolism is superior to hepatic fructose metabolism in animals other than mice. The relative importance of intestinal fructose metabolism may vary between species [25]. It is remarkable that the expression level of GLUT5 on the hepatocyte membrane is low under physiological conditions, fructose is mainly transported through GLUT2, and possibly GLUT8 is involved in the uptake of fructose in hepatocytes [26]. KHK catalyzes the formation of F-1-P from fructose entering hepatocytes. F-1-P is hydrolysed by aldolase B to glyceraldehyde (GA) and dihydroxyacetone phosphate (DHAP), and aldolase B deficiency leads to hereditary fructose intolerance (HFI) [27]. GA is further phosphorylated by triokinase to form glyceraldehyde-3-phosphate (GA-3-P). Both intermediates, DHAP and GA-3-P, can directly enter the glycolysis pathway for further metabolism. They can generate pyruvate and enter the tricarboxylic acid (TCA) cycle or produce lactate and can also be converted into glycogen through the gluconeogenic pathway. GLUT5, KHK, aldolase B and trikinase are key factors in the fructose catabolic pathway, and their activity in the intestine and liver increases with higher dietary fructose level [28]. Fructose can also be directly phosphorylated to fructose-6-phosphate (F-6-P) by hexokinase (HK) IV in hepatocytes [29]. Nevertheless, HK IV has a low affinity for fructose ( *K*_m_>100 mM) and therefore forms little F-6-P in the liver, while high level of glucose also competitively inhibits fructose phosphorylation. Small intestinal epithelial cells metabolize approximately 12% of fructose in a similar manner [30]. 

Fructose feeding increased the expression of fructolysis- and gluconeogenesis-related enzymes in the small intestine of adult wild-type (WT) mice. However, this promotion was not observed in *SLC2A5* ^*-*/ *-*^ or *KHK* ^*-*/ *-*^ mice [31]. Since fructose catabolism bypasses the negative feedback regulation of GA-3-P in glycolysis, several metabolic intermediates can rapidly accumulate *in vivo*. Continuous and high-throughput fructose metabolism, which is not controlled by the cellular energy state, leads to increased adipogenesis and increased uric acid production due to rapid ATP consumption, which are important reasons for the occurrence and development of metabolic diseases [ 32– 34]. It is worth noting that fructose may be the only carbohydrate that can produce uric acid, and reducing fructose intake has been suggested as a primary dietary change in the treatment of hyperuricemia [35]. In addition, excessive fructose intake induces excessive production of reactive oxygen species (ROS) in the nucleus tractus solitarius (NTS) of the rat brain, which in turn causes hypertension, which is alleviated after downregulation of the expression of NTS GLUT5 [36]. Additionally, GLUT5 is expressed in preadipocytes, which is associated with visceral obesity caused by fructose intake [37]. This suggests that GLUT5 is responsible for almost all of the physiological and pathological effects of fructose. 

## Physiological Expression Regulation of GLUT5 in Mammals

Each GLUT transporter has unique patterns of tissue distribution and gene regulation. Human GLUT5 is highly expressed on the apical and basolateral membranes of small intestinal epithelial cells [ 38– 40] and at low levels in cells and organs such as red blood cells, kidneys, sperm, adipose tissue, muscles, and brain [ 41 – 43]. The physiological expression and activity of human GLUT5 are the highest in the proximal duodenum and gradually decrease along the small intestine from proximal to distal regions [44]. However, in bovines, GLUT5 mRNA is the most abundant in the liver and kidney [45]. As mentioned above, GLUT5 is expressed in preadipocytes, but GLUT5 is not expressed in mature adipocytes, suggesting that GLUT5 may be involved in the development or differentiation of adipocytes [37]. The levels of GLUT5 mRNA in the intestines are barely detectable in humans and rats at birth, and only low levels are expressed during the entire lactation period (0–14 days for rats) and weaning period (14–28 days for rats), and the GLUT5 mRNA abundance and activity increase significantly after weaning (>28 days in rats) [46]. Incredibly, neonatal rats are insensitive to fructose during 0–14 days, and fructose feeding does not induce small intestinal GLUT5 expression; only rats above 14 days of age respond to intestinal luminal fructose stimulation [47]. What causes GLUT5 expression in the intestines of newborn pups to respond so significantly to fructose stimulation during lactation and weaning? Early studies have shown that the physiological response of intestinal cells to fructose in neonatal rats and newborns is regulated by glucocorticoids [ 48, 49]. The authors used the glucocorticoid analogue dexamethasone to artificially stimulate the intestines of lactating pups and observed a rapid and significant increase in GLUT5 expression level [49]. In addition, the presence of thyroid hormone response elements was found in the –338/–272 bp promoter region of the GLUT5-encoding gene, implying that thyroid hormone is likely involved in the regulation of physiological expression of GLUT5, although this role is not clear [ 50, 51]. In addition, GLUT5 expression in adult rats also showed a distinct circadian rhythm and was independent of fructose intake [52]. Recently, Zwarts *et al*. [53] demonstrated that liver X receptor α (LXRα) is capable of regulating the human and mouse *GLUT5* promoters, and the presence of LXR response elements was found at the human GLUT5 promoter relative to the transcription initiation site –385 bp. However, more detailed and in-depth regulatory mechanisms need to be further investigated. 

GLUT5 expression may be regulated more by intestinal luminal fructose signaling than by endocrine signaling. The dietary fructose-induced increase in GLUT5 expression involves the *de novo* synthesis of the corresponding mRNAs and proteins [ 54 , 55]. Carbohydrate response element binding protein (ChREBP) mediates dietary fructose-induced transcription of the *SLC2A5* gene, a basic helix-loop-helix/leucine zipper transcription factor that is highly expressed in intestinal epithelial cells and plays a critical role in the control of the expressions of genes related to glycolysis and lipogenesis [ 56, 57]. Both dietary glucose and fructose can activate the translocation of ChREBP from the cytoplasm to the nucleus, where it forms heterodimers with Max-like protein X (MLX) in the nucleus and then combines with target genes containing carbohydrate response elements (ChoREs) [ 58, 59]. High-fructose feeding systemic or intestinal-specific knockout ChREBP mice cannot induce GLUT5 expression and exhibit malabsorption syndrome (mainly characterized by diarrhea, weight loss, and intestinal distention), impaired metabolism, decreased body temperature and even near death within 1–2 weeks [ 60, 61]. Consistent with this, mice with hepatic ChREBP deficiency do not exhibit fructose intolerance [62]. It was experimentally demonstrated that the ChREBP-MLX heterodimer binds directly to ChoRE, located 2 kb from the *SLC2A5* gene (2149–2165), thereby regulating *SLC2A5* transcription, and that this protein-DNA interaction is induced by diet [61]. 

The experimental results showed that when a fructose solution was infused into the intestinal cavity of adult wild-type mice, the mRNA and protein levels and activity corresponding to GLUT5 were observed to increase by 2–10 folds [63]. However, in mice with targeted deletion of Rab11a in small intestinal epithelial cells, fructose feeding failed to induce GLUT5 expression in the small intestine, and the mice exhibited malabsorption syndrome, suggesting that Rab11a is one of the factors regulating GLUT5 expression [63]. In addition, *SLC2A5* knockout mice were able to survive normally and give birth, but after being fed with a high fructose diet, they also had hypotension and malabsorption syndrome [64]. Incredibly, even primary intestinal cells cultured *in vitro* were able to be induced to express GLUT5 by fructose [65]. Interestingly, a recent study suggests that intestinal GLUT5 expression level may also be associated with lipid intake [66]. This implies that not only saccharides but also other types of nutrients may be involved in the regulation of GLUT5 expression. 

In addition to diet, exercise also affects GLUT5 expression level. Studies have revealed that long-term running exercise can increase GLUT5 protein expression level in mouse hippocampal microglia, promote microglial glucose metabolism and improve the cognitive function of mice [67]. The regulation of GLUT5 expression is multifactorial and multifaceted. 

Thioredoxin-interacting protein (TXNIP), another fructose-inducible protein regulated by ChREBP, is expressed in key metabolic tissues such as the liver and intestine [ 68, 69]. TXNIP promotes the localization of hexose transporters to the plasma membranes, including assisting GLUT5 localization to the apical membranes of enterocytes [70]. Therefore, factors regulating GLUT5 localization and functional execution may also be important factors affecting dietary fructose absorption in the intestines [71]. 

## Structural Characteristics of the Mammalian GLUT5 Protein

GLUT transporters belong to the sugar transporter subfamily of the major facilitator superfamily (MFS). A common structural feature of the MFS members is that they all share the MFS-fold structure. The mammalian GLUT5 protein has a typical MFS folding structure: twelve hydrophobic transmembrane (TM) α-helices constitute four trimeric substructures, which in turn form two mutually separated TM bundles, namely, an N-terminal six TM bundle (TM1–6) and a C-terminal six TM bundle (TM7–12). The two six TM bundles are mirrored by rotation of approximately 180° around a false bisymmetry axis that passes through the center of the transporter and is perpendicular to the plasma membrane plane [72]. The 12 TMs are connected by hydrophilic loops of different lengths, and a large cytoplasmic ring separates the two six TM bundles between TM6 and TM7 [73]. There are some clever regular connections between different TMs, such as TM1–3 having sequence similarity with reverse TM4–6, while TM7–9 has sequence similarity with reverse TM10–12, which may be caused by gene duplication and fusion [74]. In addition, the rat and bovine GLUT5 proteins, which share 81% sequence identity with the human GLUT5 protein, have an intracellular portion that includes five helical structures, one at the C-terminus and four others located between the N-terminal six TM bundle and the C-terminal six TM bundle [75]. 

**Figure 2:**
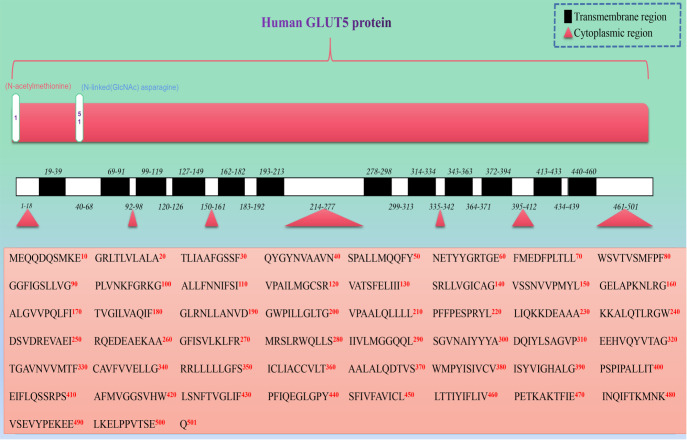
Figure 2 Human GLUT5 structure diagram queried from the Uniprot protein database The lower part of the figure shows the complete amino acid sequence of the human GLUT5 protein, with different letters representing different amino acid classes. The cytoplasmic region of the GLUT5 protein and the distribution characteristics of the 12 TMs along the amino acid sequence are indicated by red triangles or black squares, respectively.

**Figure 3:**
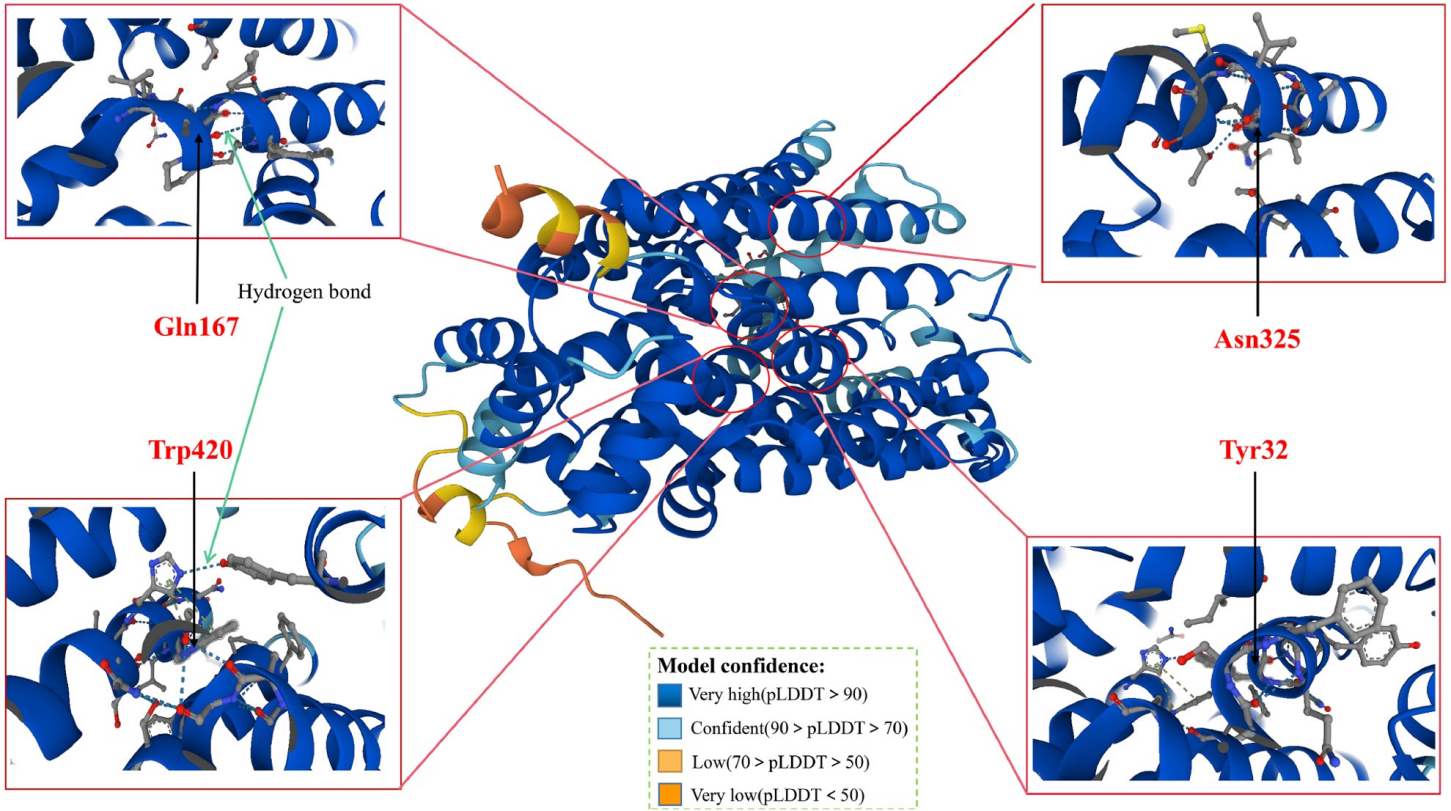
Figure 3 Diagram of human GLUT5 protein molecular structure prediction model downloaded from AlphaFold Protein Structure Database Three amino acid residues (Gln167, Asn325, Trp420) located in the central lumen region of the GLUT5 protein and one (Tyr32) facing the central lumen region were selected as representatives and their structural models were shown [76,77]. pLDDT: predicted local distance difference test.

The human GLUT5 protein expressed *in vivo* comprises a total of 501 amino acids (residues). By querying the protein database UniProt ( https://www.uniprot.org/), the distribution characteristics of the 12 TMs of the human GLUT5 protein (UniProt ID: P22732) along the amino acid sequence, as well as sites where posttranslational modifications occur on the polypeptide chain, were determined ( Figure 2). In addition, we downloaded the human GLUT5 protein molecular structure prediction model diagram ( Figure 3) from the AlphaFold Protein Structure Database ( https://alphafold.ebi.ac.uk/entry/P22732), and four different colors represent the confidence score (pLDDT) of each residue [ 76, 77].

How does the GLUT5 protein molecular structure affect the process of its binding and transport of fructose? Previous studies of GLUT5-GLUT3 chimeras have shown that two large regions containing amino acid sequences are important for the fructose transport function of GLUT5: the region between the N-terminal and the first cytoplasmic loop and the region between the third extracellular loop and TM11 [78]. However, the authors were not able to further analyze the role of individual amino acid residues. The crystal structures of GLUT5 in rat and bovine have been analyzed by X-ray diffraction. The GLUT5 substrate binding site is located in the central cavity between the N-terminal six TM bundle and the C-terminal six TM bundle, and the amino acid residues arranged here are related to substrate binding activity. The amino acid residues Gln166 (corresponding to Gln167 in human GLUT5), Ile169 (170), Ile173 (174), Gln287 (288), Gln288 (289), Asn324 (325) and Trp419 (420) are located in the central cavity, and Tyr31 (32), His386 (387), Ala395 (396), His418 (419), and Ser391 (392) also face the central cavity [75]. Trp388 and Trp412 residues are critical for the transport activity of GLUT1 [79], and Trp419 is the only tryptophan located at the central cavity substrate binding site in rat GLUT5 [75]. As measured by tryptophan fluorescence quenching, a sharp disappearance of the fluorescence intensity of tryptophan residues in the central cavity was observed when the added substrate was D-fructose. However, when the substrate was added as other monosaccharides, there was no significant change in the fluorescence intensity of the tryptophan residue in the central cavity, which represents the substrate binding activity of GLUT5 to D-fructose [75]. In addition, alanine fixed-point mutants of Tyr31, His386, His418, Ser391, and Ala395 in human GLUT5 substrate binding sites all lead to strong substrate binding activity weakening, and the rest of the amino acid residues except Tyr31 belong to the C-terminal six TM bundle, indicating that the N-terminal six TM bundle and the C-terminal six TM bundle in GLUT5 are asymmetrically bound to fructose [75]. Single amino acid mutations Y31F (indicating that the Tyr mutation at site 31 is Phe), Q166E, Q287A, H386F, H386A, S391A and H418Q lead to a significant reduction in fructose binding of GLUT5 protein (<40% of wild-type GLUT5 protein), while the fructose binding of GLUT5 protein caused by Y31A, Q166A, I169A, I173A and Q288A is smaller (approximately 40%‒90% of wild-type GLUT5 protein) [75]. 

**Figure 4:**
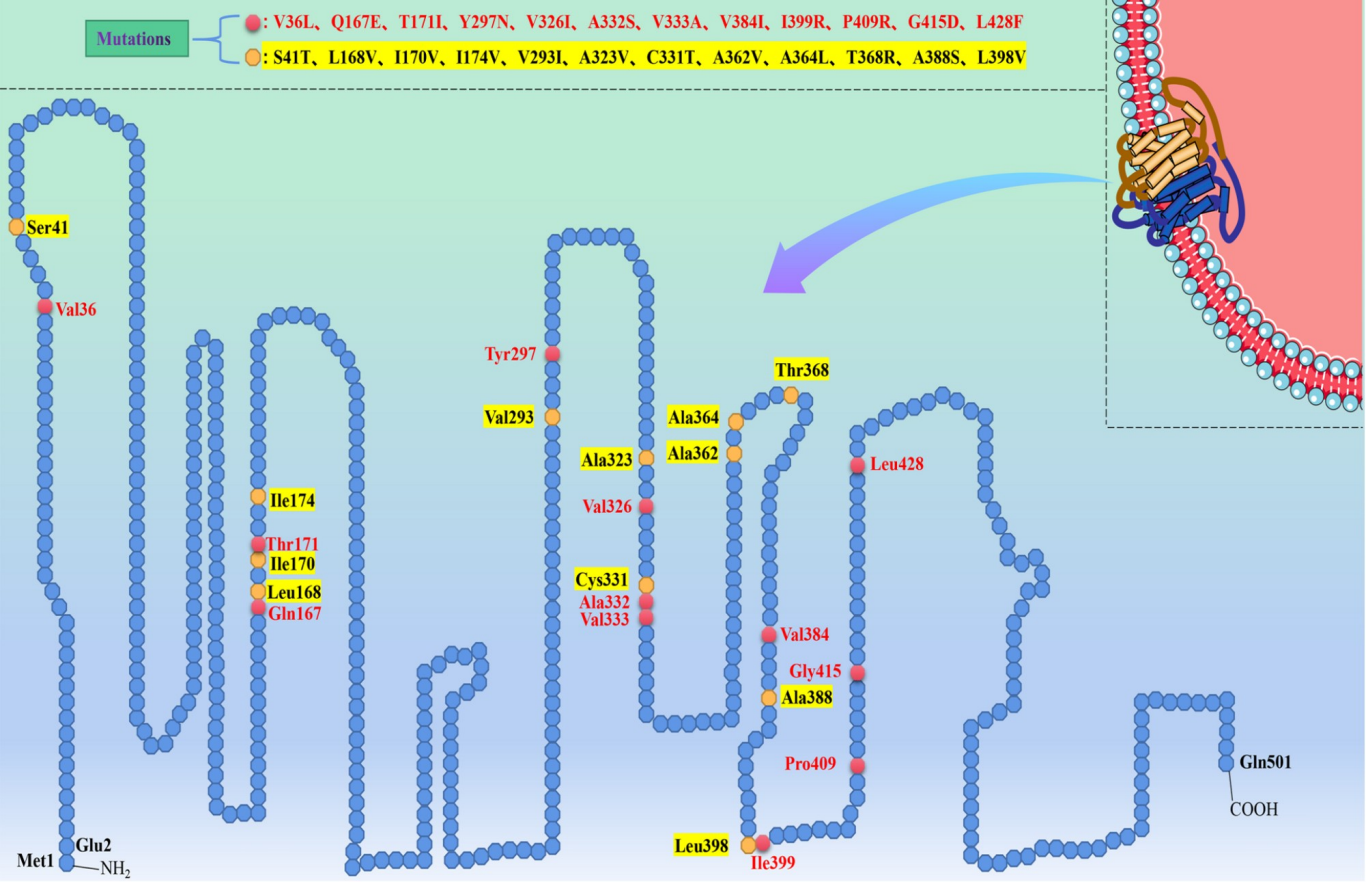
Figure 4 Diagram of single amino acid mutation in the amino acid sequence of human GLUT5 protein Each regular octagon represents an amino acid residue. The red regular octagons represent an amino acid mutation that reduces the amount of fructose transported by chimeras to less than 30% of the wild-type GLUT5 protein, while the yellow regular octagons represent a 30% to 80% reduction [81].

GLUT7, also expressed in the small intestine, also belongs to class II GLUTs, and its protein sequence has 512 amino acid residues, which is approximately 60% similar to GLUT5, but has no ability to transport fructose [80]. The GLUT5-GLUT7 chimera is considered a suitable model to investigate the role of individual amino acid residues in fructose recognition and transport of GLUT5. Ebert *et al* . [81] divided the protein sequence of human GLUT5 into 26 fragments and consecutively replaced these fragments with homologous domains of GLUT7 to obtain GLUT5-GLUT7 chimeras F1‒F25. They found that fructose intake in chimeras F2 (23–41), F13 (242–254), F17 (323–338), F18 (343–357), F19 (361–381), and F25 (488–501) was reduced by 30 to 80% compared with wild-type GLUT5 protein. In contrast, several other chimeras, F9 (164–181), F15 (286–305), F20 (382–399), and F21 (409–428), showed fructose intake even lower than 30% of wild-type GLUT5 fructose intake [81]. However, the chimeric fragments containing multiple amino acid residues remain insufficiently precise. The authors further divided each chimera into fragments containing fewer amino acid residues and found that single amino acid mutations at sites 36, 167, 171, 297, 326, 332, 333, 384, 399, 409, 415, and 428 resulted in lower fructose transport in chimeras than 30% of fructose transport in the wild-type GLUT5 protein, while single amino acid mutations at sites 41, 168, 170, 174, 293, 323, 331, 362, 364, 368, 388 and 398 resulted in a 30% to 80% decrease in fructose transport in the chimer [81] ( Figure 4). These amino acids, which are important for GLUT5 fructose transport function, are present in the first extracellular loop, TM5, TM7, TM8, TM9, TM10, and the region between TM9 and TM10, TM10 and TM11, respectively [81].

Past studies have found that substrate transport by MFS proteins involves the continuous destruction and formation of some inter-TM bundle salt bridges in the central lumen of proteins [82]. In rat GLUT5, Glu151 and Arg97 in the N-terminal six TM bundle formed an inter-TM bundle salt bridge with Arg407 in the C-terminal six TM bundle, and similarly, Arg158 in the N-terminal six TM bundle formed an inter-TM bundle salt bridge with Glu400 and Arg340 in the C-terminal six TM bundle. In addition, Glu336 in the C-terminal six TM bundles is connected to the inter-TM bundle salt bridge by forming an intra-TM bundle salt bridge with Arg340. The network of salt bridges formed by these salt bridges spanning multiple TM bundles plays a role in maintaining the external conformational stability of the GLUT5 protein [75]. 

## Structural Mechanisms of Substrate Recognition and Transport of Mammalian GLUT5 Protein

**Figure 5:**
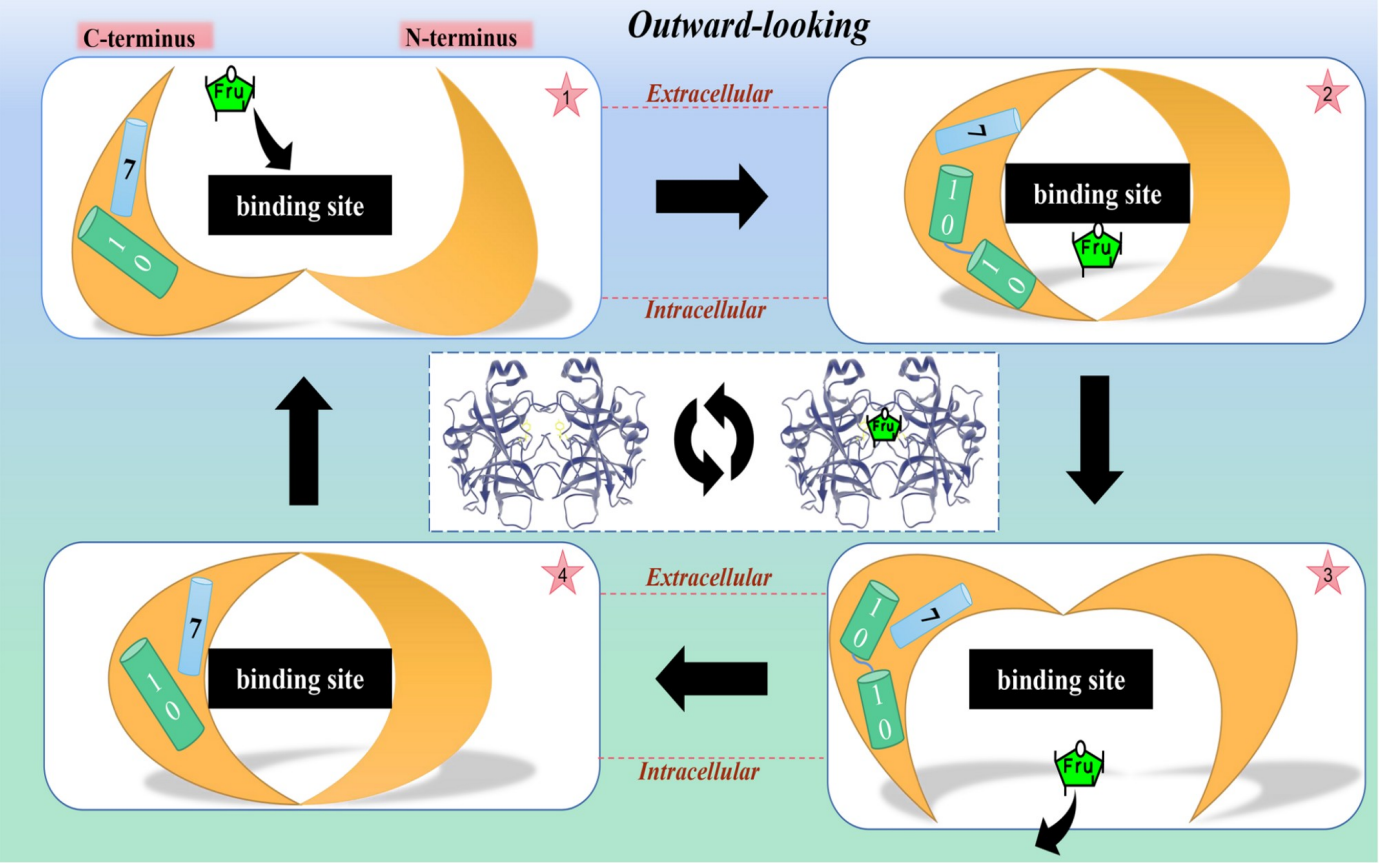
Figure 5 Mechanism diagram of the binding and transport of fructose by GLUT5 A complete cycle involves the fructose binding to the fructose removal process. The rigid body motion of the two six-TM bundles constitutes the “rocker switch” motion control, and the interaction of TM7 and TM10 locally forms the gating mechanism. Over the course of the cycle, TM10 has undergone structural changes [75,84,85 ].

Alternating access transport mechanisms have long been the most commonly used model to explain transporter substrate transport mechanisms. During a transport cycle, the transporter undergoes transient conformational changes of outwardly open (unloaded), outwardly closed (substrate binding), inwards open (substrate release), inwards closed (unloaded), and outwardly open (unloaded), with the four major conformations alternately exposed to each side of the membrane to transport and release the substrate through the lipid bilayer [83]. The symmetrical binding of the N-terminal and C-terminal six TM bundles to the substrate around the central cavity substrate binding site and the rigid body motion of the two six TM bundles form the structural basis of the MFS-type ″rocker switch″ mechanism and thus complete alternating channel transport [ 84, 85]. The substrate transport of GLUT5 may not only be controlled by the ″rocker switch″ motion of the N- and C-terminal six TM bundles, as its two six TM bundles display asymmetric binding to the substrate. By analyzing the crystal structure of rat and bovine GLUT5, Norimichi Nomura and his team [75] further proposed that TM7 and TM10 in the C-terminal six-TM bundle perform gating movements locally through interactions and couple with substrate binding and release based on experiments analyzing the crystal structures of GLUT5 protein in rats and cattle ( Figure 5). In addition, there is a highly conserved salt bridge motif, RXGRR, between the cytoplasmic loops of TM2 and TM3, which is repeated between the cytoplasmic loops of TM8 and TM9, and these salt bridge sequences are associated with conformational changes that occur during substrate transport [86].

Although the *K*_m_ value of GLUT5 for D-fructose varies in different species, D-fructose is always the optimal substrate. Although Kishida *et al*. [87] demonstrated that GLUT5 in the small intestine transports D-allulose, an epimer of D-fructose, its affinity for D-allulose is much lower than that of D-fructose. Therefore, how does GLUT5 specifically identify fructose? In aqueous solution, both glucose and fructose exist in the conformation of pyranose or furanose, with 99% of glucose being pyranose and 31.5% of fructose being furanose [88]. In the protein crystal structure bound to glucose, glucopyranose is the predominant form; in the protein crystal structure bound to fructose, furanose is the predominant form (Protein Data Bank, https://www.rcsb.org/). GLUT5 identifies furanose and pyranose conformations of fructose, binding involving interactions with fructose at positions C _1_, C _2_, C _3_ and C _4_ [89]. Furthermore, amino acid residues not only play a role in substrate binding activity but are also essential structural bases for substrate recognition by GLUT5. For example, GLUT1, 3, and 4, which transport only glucose, have QLS sequences in TM7, a region that is capable of interacting with the C _1_ site of D-glucose and is closely involved in substrate recognition by the substrate binding site on the outer surface; TM7, which transports glucose and fructose, contains the HVA sequence, while the corresponding region of GLUT5, which specifically transports fructose, is the MGG sequence [90]. 

## GLUT5 Inhibitor

In-depth analysis of the GLUT5 crystal structure and substrate recognition-transport mechanism is instructive for designing specific inhibitors. Inhibitors of GLUTs interfere with the entire transport process mainly by hindering the transitions of different conformations of transporters during the transport cycle or inhibiting the binding of substrates to transporters [91]. Although there is high sequence similarity among GLUT members at the gene and protein molecular levels, some known GLUT protein inhibitors (such as cytochalasin B, phloretin, or forskolin) do not inhibit GLUT5-specific fructose transport. Earlier studies have identified several natural inhibitors of GLUT5, such as green tea catechins [92] and rubusosides from Chinese sweet tea extraction [93]. However, these natural inhibitors are nonspecific and have low binding inhibitory efficacy (IC _50_ is ~5 mM). Currently, computer high-throughput ligand screening methods have been applied to develop novel GLUT5 inhibitors with therapeutic potential [94]. For example, N-[4-(methylsulfonyl)-2-nitrophenyl]-1,3-benzodioxol-5-amine (MSNBA), which is specific to GLUT5 and does not affect the fructose transport capacity of human GLUT2 or the glucose transport capacity of human GLUT1-4, is currently known as the most effective GLUT5-specific inhibitor [95]. In-depth studies have shown that the MSNBA binding site is close to the active center of the GLUT5 protein and is responsible for substrate recognition by residue H387, as well as residues such as Ser143, Thr171, Gln288, Gln289, Asn294 and Tyr297 involved in the binding process of MSNBA-GLUT5 [95]. Recently, researchers have found that some phenolic-rich dietary plant extracts can reduce the transcript levels of the gene encoding ChREBP, thereby inhibiting GLUT5 protein expression and its mediated fructose transport [96]. This may contribute to our better understanding of the regulation of GLUT5 expression by plant compounds present in the human diet. The development of novel GLUT5 inhibitors is significant for the treatment strategy of diseases related to fructose metabolism. It is not the focus of this article, and we will not discuss it in depth. Readers can refer to the review published by Reckzeh *et al*. [10]. 

## Relationship between GLUT5 and Intestinal Diseases

The role of GLUT5 in intestinal system diseases has raised widespread concern as a result of its decisive role in intestinal fructose absorption. The abnormal expression of GLUT5 may be an important factor in the pathogenesis of certain digestive system diseases.

### Gut microbiota dysbiosis and intestinal barrier impairment

The microbiota that inhabit the gut is the largest and most complex microbial community in the human body, consisting of bacteria, fungi, viruses, archaea and protozoa. The interaction between the gut microbiota and the human physiological internal environment is a principal element influencing host development, metabolism, the intestinal barrier and innate immunity [ 97, 98]. There is increasing evidence that metabolic disorders are associated with the normal gut microbiota and intestinal barrier impairment [ 99– 101]. The metabolic capacity, community size and community composition of the gut microbiota may change with the dietary fructose level of the host, thus resulting in adaptation [102]. However, sustained and high fructose stimulation can trigger gut microbiota dysbiosis, which can lead to the development of disease. For instance, Li *et al*. [103] identified that feeding with a high-fructose diet for 8 weeks induced intestinal microbiota imbalance, short-chain fatty acid reduction, and intestinal epithelial barrier damage, and the more severe consequences were the hippocampal neuroinflammatory response, reactive gliosis, and neuronal loss in C57BL/6 N mice. It was reported that feeding with 10% fructose for 7 days exacerbated the manifestations of colitis induced by 2.5% dextran sulfate sodium (DSS) in mice, such as diarrhea, ulcers, rectal bleeding and colon shortening [104]. The analysis showed significant changes in β-diversity ( *P*<0.001) but not in α-diversity ( *P*=0.06) of the intestinal microbial community of mice. Higher levels of Clostridium cluster IV and Enterococcus sp. were found in the feces of mice with intestine-specific GLUT2 deletion (GLUT2 ^ΔIEC^) and overexpression of GLUT5 [105]. Thus, changes in dietary fructose level and intestinal GLUT5 expression can contribute to the development of disease by altering the normal intestinal microbiota status and intestinal barrier function. 

### Inflammatory bowel disease

Inflammatory bowel diseases (IBDs) are a group of chronic intestinal diseases, mainly ulcerative colitis (UC) and Crohn′s disease (CD), which have become global diseases in the 21st century [106]. A clinical study showed that GLUT5 was expressed in the brush border membrane of large intestinal mucosal epithelial cells in IBD patients (UC, *n*=18; CD, *n*=10) and may be involved in the formation of abnormal lymphatic vessels in the lamina propria, since GLUT5 labeling was also observed in abnormal lymphatic vessel clusters, which is a new characteristic histological finding in the pathogenesis of IBDs [107]. The team continued to examine the expressions of leptin and leptin receptor in the large intestine mucosa of patients with IBDs in the same biopsies and found that leptin and leptin receptor immunolabelling localization was shown in the subepithelial structure of the lamina propria of the large intestine, and GLUT5 immunoreactivity was identified in specific lamina regions expressing leptin and leptin receptors [108]. This suggests a possible link between fructose and the leptin system and promotes the formation and growth of blood vessels and lymphatic vessels in the lamina propria of the large intestine in IBD patients through GLUT5. Angiogenesis and lymphangiogenesis are hallmark features of chronic intestinal inflammation, and the dilation of these vascular groups may play a pathogenic role in IBDs [109]. Changes in dietary fructose and GLUT5 may play important roles. 

High fructose in modern diets is a key factor in the rising incidence and exacerbating the progression of IBDs. One of the reasons is that high fructose diet feeding can reduce the thickness of colonic mucus and alter the composition and metabolism of the gut microbiota [110]. In a recent study, *SLC2A5* ^+/+^ and *SLC2A5* ^–/–^ mice fed with a 15 Kcal% fructose diet were found to exhibit more severe DSS-induced experimental colitis [111]. This effect is associated with increased level of free fructose in the colon and changes in the fecal microbiota in *SLC2A5* ^–/–^ mice, and broad-spectrum antibiotics can prevent the worsening of colitis in *SLC2A5* ^–/–^ mice [111]. This is consistent with the conclusion that changes in GLUT5 expression lead to changes in the gut microbiota, as mentioned earlier. In conclusion, targeting abnormal GLUT5 expression in the large intestine may be an effective means of alleviating or treating IBDs in the future. 

### Colorectal cancer

Colorectal cancer (CRC) is one of the major causes of death among cancer patients worldwide [112]. CRC cells exhibit hyperactive glycolysis, and the consumption of massive glucose leads to glucose deficiency in the tumor microenvironment (TME) [113], which means that CRC cells may have alternative energy sources. In fact, an earlier study showed that GLUT5 expression was detected in colorectal tissue samples from both healthy individuals and CRC patients [114]. Recently, GLUT5 mRNA expression was detected in 96.7% of cancer tissue samples from 30 patients at different stages of CRC, with a significant positive correlation between GLUT5 expression level and cancer grade. The GLUT5 mRNA expression level was almost 2.5- *folds* higher in the colonic mucosa of CRC patients than in the colonic mucosa of non-CRC controls ( *P* <0.001) [115]. Moreover, treatment with the GLUT5-specific inhibitor MSNBA for 24 h significantly decreased the viability of the human CRC cell line HT-29 (51% reduction at 10 ìM and 55% reduction at 1 ìM) but had a minimal effect on CCD 841 CoN in the human normal colonic epithelial cell line (8% reduction at 10 ìM and 2% reduction at 1 ìM) [115]. In addition, it was reported that CRC cells highly expressing GLUT5 exhibited significant fructose-induced proliferation in a glucose-deficient but fructose-enriched culture environment [116]. However, the addition of fructose did not affect the proliferation rate of CRC cells when the culture environment was enriched with glucose. These results suggest that fructose is an important alternative energy source to promote the proliferation of CRC cells with high GLUT5 expression when glucose level in the TME is reduced. This may explain why CRC cells are able to be induced by fructose to highly express GLUT5 under hypoxic conditions, which in turn improves survival [117]. 

Although little is currently known about the role of GLUT5 in CRC cell metastasis and invasion, some encouraging findings have been published. Lin and his team [118] reported that GLUT5 mRNA and protein showed high expressions in human CRC tumor tissues compared to adjacent normal tissues, and the protein was expressed both on the cell membrane and in the cytoplasm. Further assays demonstrated that *SLC2A5* overexpression promotes CRC cell invasion and migration *in vivo* and *in vitro*, while knockdown of *SLC2A5* showed opposite results. These results stimulated the team’s interest in further exploring whether high expression of GLUT5 is associated with the epithelial-mesenchymal transition (EMT) process in CRC cells, as EMT has long been shown to be associated with the capacity of malignant cells to metastasize and invade [119]. As expected, the morphology of CRC cells overexpressing GLUT5 changed from an epithelial-like form to a spindle-shaped or elongated mesenchymal form, and the expressions of the EMT-related markers N-cadherin and vimentin were significantly upregulated [119]. This suggests that GLUT5 can promote CRC cell metastasis and invasion by inducing EMT. 

One of the main reasons why cancer is difficult to treat is that malignant cells become resistant to chemotherapy drugs, which makes it impossible for all types of chemotherapy drugs to destroy all malignant cells. A concurrent study by Shen *et al*. [116] also discovered that *SLC2A5* gene knockdown can significantly reduce the resistance of CRC cells to the chemotherapeutic drug oxaliplatin. A recent study came to a similar conclusion that decreased expression of the *SLC2A5* gene caused CRC cells to be sensitive to cisplatin or oxaliplatin [120]. Furthermore, the use of the fructose analogue 2,5-anhydro-D-mannitol (2,5-AM), which hinders GLUT5 transport fructose, greatly improves the elimination of oxaliplatin to malignant cells. In another work, researchers observed that GLUT5 expression was enhanced at both the mRNA and protein levels in CRC cells stably resistant to oxaliplatin and 5-fluorouracil [121]. Following knockdown of *SLC2A5* in drug-resistant CRC cells, cancer cells exhibited significantly reduced expression of enzymes related to glycolysis and lipogenesis, resulting in reductions in lactate and fatty acid levels and NADP/NADPH ratios. Furthermore, targeted inhibition of GLUT5 also prevented the migration and invasion of chemoresistant CRC cells [121]. 

These studies demonstrate the important role of GLUT5 in CRC survival metabolism, metastasis, invasion and drug resistance. Therefore, targeting GLUT5 synergistic chemotherapy drug treatment may be a potential strategy to inhibit CRC growth and metastasis in the future.

### Fructose malabsorption

Fructose malabsorption (FM) is a common digestive disorder that is common in infants and patients with gastrointestinal diseases and is associated with impaired absorption of fructose from the small intestine [ 122, 123]. Clinically, patients with FM will exhibit typical gastrointestinal symptoms with irritable bowel syndrome (IBS), such as diarrhea, abdominal distention, and abdominal pain [124]. Indeed, approximately one-third of IBS patients are diagnosed with FM manifestations, whereas restricted fructose intake is able to alleviate their clinical symptoms [ 125– 127]. Abnormalities in the major fructose transporters in the intestines are considered to be responsible for the development of FM [61]. In a previous paper, we described that ChREBP, Rab11a, and GLUT5 deletions cause intestinal FM in mice, which in turn leads to malabsorption syndrome manifestations [ 60– 64 ], and we will not repeat them here. In particular, clinical samples showed that duodenal GLUT5 mRNA and protein expressions did not differ significantly between adult FM patients ( *n*=11) and healthy people ( *n*=15) [128], suggesting that adult FM may not be significantly related to intestinal GLUT5 expression. A recent study published by Staubach *et al*. [129] also supports this conjecture. In contrast, infants with high fructose intake are more likely to develop FM, which may be due to low intestinal GLUT5 expression and activity rather than *SLC2A5* gene mutation [130]. 

The GLUT5-related intestinal diseases described in this paper are summarized in Table 2.

**Table:** **Table 2** GLUT5-associated intestinal diseases and the role of GLUT5 in these diseases

Disease	Role of GLUT5	Reference
Gut microbiota dysbiosis/gut barrier dysfunction	Changes in dietary fructose levels and intestinal GLUT5 expression contribute to disease development by altering normal gut microbiota and gut barrier function.	[ 97– 105]
IBDs (including UC and CD)	GLUT5 promotes the formation and growth of blood vessels and lymphatic vessels in the lamina propria of the large intestine in patients with IBDs.	[ 106– 111]
CRC	High expression of GLUT5 promotes CRC cells proliferation, metastasis, invasion and enhances drug resistance of cancer cells.	[ 112– 121]
FM	Fructose malabsorption due to lack of ChREBP or rab11a, or low expression of GLUT5 itself.	[ 61, 122– 130]

## Upregulated GLUT5 Promotes the Progression of Multiple Cancers

The expression of GLUT12, a nonspecific glucose and fructose transporter belonging to class III GLUTs, has been suggested as a possible therapeutic target for early and advanced breast cancer [131]. In addition to CRC, GLUT5 has been found to be closely associated with other malignancies in recent years. For example, the expression of GLUT5 was significantly higher in glioma cells than in normal glial cells and was significantly correlated with the malignancy of glioma and the low survival rate of glioma patients ( *P*<0.01). GLUT5 expression downregulation could significantly inhibit tumor proliferation *in vivo* [132]. Furthermore, the upregulation of GLUT5 expression in ovarian cancer tissues was significantly associated with tumor malignancy and poor survival in ovarian cancer patients, and silencing of *GLUT5* in ovarian cancer cells significantly inhibited tumor cell proliferation and migration [133]. Weng and her team [134] found that the expression of the *SLC2A5* gene is upregulated in lung adenocarcinoma (LUAD) patients and is highly associated with poor prognosis in lung adenocarcinoma patients. Overexpression of *SLC2A5* enhances LUAD cell proliferation, migration, invasion, and tumorigenicity in fructose-containing culture medium, and cancer cells are more sensitive to paclitaxel treatment after inhibition of GLUT5 with 2,5-AM. Chen *et al*. [135] further demonstrated the importance of GLUT5-mediated fructose utilization *in vivo* in regulating LUAD growth. In addition, the *SLC2A5* gene and its encoding GLUT5 are upregulated in malignant tumors such as prostate cancer, breast cancer, acute myeloid leukemia and clear cell renal cell carcinoma and promote tumor progression [ 136– 139]. 

What exactly is the role of GLUT5 in cancer progression? Normal cells tend to acquire new metabolic pathways after malignant transformation into cancer cells. In contrast, cancer cells are more prone to utilize fructose as a source of metabolic raw material. Numerous previous studies have confirmed the preferred utilization of fructose in multiple cancers of multiple systemic origins and have been associated with upregulation of GLUT5 expression [140]. In human nonproliferating cells, fructose is mainly metabolized by KHK. However, cells such as cancer cells which have strong proliferative capacity, usually transform KHK-c with a high affinity for fructose into KHK-a with a low affinity [141]. Moreover, the expression level of HK in these proliferating cells is significantly higher than that of KHK-a, which may precisely meet the needs of new metabolic pathways. In fact, cancer cells only need to stably overexpress GLUT5 protein to promote their own proliferation by metabolizing fructose, but unlike traditional understanding, this phenomenon may have little to do with KHK, which is mainly responsible for metabolizing fructose [142]. Suwannakul and her colleagues [143] provided strong evidence for the idea that GLUT5 promotes fructose metabolism in cancer cells. They found that cell proliferation and ATP production were significantly increased in cholangiocarcinoma (CCA) cells that highly expressed GLUT5, particularly in medium supplemented with fructose. Conversely, silencing of *GLUT5* caused decreased CCA cell proliferation and ATP production and attenuated cell migration and invasion [143]. 

In addition to metabolic pathways, GLUT5 is also associated with the cancer-promoting inflammatory environment, as a positive correlation between GLUT5 expression levels and the inflammatory factor interleukin-6 (IL-6) has been observed during the progression of multiple cancers. Knockdown of *GLUT5* has been reported to eliminate fructose uptake and utilization by oral squamous cell carcinoma and prostate cancer cells induced by interleukin-6 and inhibit cancer cell proliferation [144]. This suggests that there are also a series of cascade responses between fructose metabolism and the inflammatory microenvironment in cancer cells, and GLUT5 is one of the key regulators. Notably, IL-6-activated inflammatory signals are also associated with the pathogenesis of IBDs [145]. Perhaps GLUT5 is an important ″bridge″ mediating extracellular-intracellular signal transition during inflammation-induced cell carcinogenesis. 

**Figure 6:**
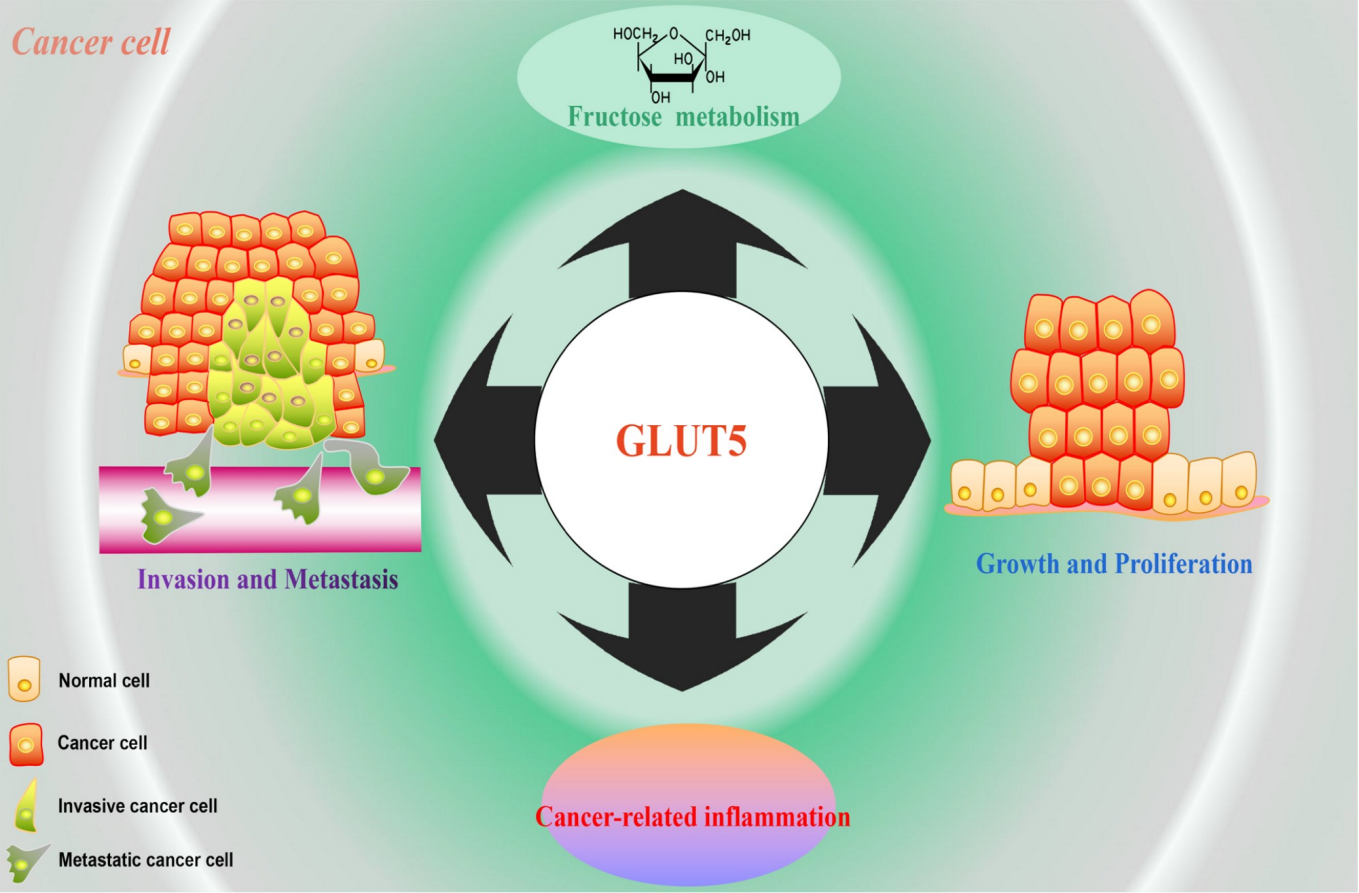
Figure 6 The GLUT5 protein promotes a varity of biological behaviors of cancer cells [ 116 – 121 , 132– 144]

In conclusion, the fructose-GLUT5 axis is indeed an important driver of a variety of biological behaviors of cancer cells ( Figure 6), just as excessive fructose uptake promotes metastasis of CRC cells to the liver or enhances nucleotide synthesis in pancreatic cancer cells [ 146 , 147]. Considering the special status of GLUT5 as a fructose-specific transporter and its close relationship with multiple malignancies ( Table 3), researchers have attempted to deliver bioactive agents into GLUT5 ^+^ malignant cells by using this membrane protein for the purpose of cancer treatment.

**Table:** **Table 3** Malignant tumors associated with high GLUT5 expression mentioned in this review

Cancer	Expression of GLUT5	Influence	Reference
Colorectal cancer	Up-regulated	GLUT5 promotes cancer cell proliferation, migration and invasion, and enhances cancer cell drug resistance.	[ 114– 121]
Glioma	Up-regulated	GLUT5 is associated with tumor malignancy and poor patient survival.	[132]
Ovarian cancer	Up-regulated	GLUT5 is associated with tumor malignancy and poor patient survival.	[133]
Lung adenocarcinoma	Up-regulated	GLUT5 promotes cancer cell proliferation, migration, invasion and tumorigenicity, enhances cancer cell drug resistance and is associated with poor patient prognosis.	[ 134, 135]
Breast cancer	Up-regulated	GLUT5 promotes tumor progression.	[137]
Acute myeloid leukemia	Up-regulated	GLUT5 promotes tumor progression.	[138]
Clear cell renal cell carcinoma	Up-regulated	GLUT5 promotes tumor progression.	[139]
Cholangiocarcinoma	Up-regulated	Cancer cells with high GLUT5 expression showed increased cell proliferation and ATP synthesis.	[143]
Oral squamous cell carcinoma	Up-regulated	Up-regulated GLUT5 mediates increased uptake and utilization of fructose by cancer cells induced by IL-6.	[144]
Prostate cancer	Up-regulated	Up-regulated GLUT5 mediates increased uptake and utilization of fructose by cancer cells induced by IL-6.	[ 136, 144]
Pancreatic cancer	Up-regulated	GLUT5-mediated fructose uptake and utilization promote nucleotide synthesis in cancer cells.	[147]

## Development of GLUT5-targeted Diagnostic Probes

The recognition that malignant cells have higher energy requirements for a long time [148] has led to the development of metabolic-based cancer detection methods. The differences in GLUT expression between different cells as well as the large amount of sugar consumption in cancer cells make GLUTs important therapeutic targets. Kinetic analysis of glucose and fructose uptake provides the structural basis for the development of fluorinated hexose derivatives for positron emission tomography (PET) imaging of malignant cells [149]. 18F-labelled 2-fluoro-2-deoxy-D-glucose (2-FDG) is the earliest and most commonly used radiotracer for PET [150]. 2-FDG is not completely metabolized after uptake by malignant cells, which gives rise to its continuous accumulation in malignant cells, and is widely used in clinical practice to observe glucose uptake, tumorigenesis and invasion [ 151, 152]. However, since 2-FDG mainly targets GLUT1, which is ubiquitous in tissue cells, abundant false positive results show the insufficient ability of tracers targeting the glucose transporter GLUT1 to distinguish malignant cells [153]. In particular, in breast cancer, there is no significant difference in glucose uptake between malignant and normal cells, which limits the methods of detecting targeted glucose transport [ 154 , 155 ]. Since GLUT5 expression upregulation has a significant promoting effect on cancer, people have begun to pay attention to the development of targeted probes for fructose transport. 

Fructose phosphorylation can occur at position C _1_ (KHK) or C _6_ (HK), so targeted probes designed for fructose transport mainly focus on these two sites. The first targeted GLUT5 tracer tested in tumors was the fluorinated fructose derivative 1-[18F]fluoro-1-deoxy-D-fructose (1-FDF), designed by Haradahira and colleagues [156], which showed rapid washout of 1-FDF by the kidney and liver *in vivo*. Triple-negative breast cancer cells and tissues express higher levels of GLUT5 mRNA and protein than estrogen receptor-positive breast cancer cells and tissues, and the growth and progression of breast cancer are highly dependent on fructose [157]. Thus, the 1-FDF analogue 6-[18F]fluoro-6-deoxy-D-fructose (6-FDF) was tested for PET imaging in murine EMT-6 and human MCF-7 breast cancer cells expressing GLUT5 [158]. 6-FDF has been shown to be the substrate of human KHK and is rapidly metabolized *in vivo*. In addition, PET imaging tests of 3-[18F]fluoro-3-deoxy-D-fructose (3-FDF) in EMT-6 and MCF-7 cells demonstrated that GLUT5 can transport both furanose and pyranose forms of fructose [159]. 

Fluorophore labelling with 7-nitro-1,2,3-benzadiazole (NBD) at the fructose C _1_ position (1-NBDF) was able to target breast cancer cells GLUT5 well. The absorption of 1-NBDF probes was studied in three breast cancer cell lines: MCF 7, MDA-MB-435, and MDA-MB-231. 1-NBDF showed very good absorption in all cell lines tested, with uptake levels comparable to the corresponding glucose analogue 2-NBDG. Significant uptake of 1-NBDF was not observed in cells lacking GLUT5, while GLUT5-specific accumulation was detected in cells expressing GLUT5 [160]. 

2,5-AM aryl conjugates with high affinity and specificity for GLUT5 have emerged as a new generation of radiotracer probes. 2,5-AM is a symmetric molecule that exists only as a furanose ring structure and cannot be opened to form isomers [161]. The 1-amino-2,5-anhydro-d-mannitol-NBD conjugate (NBDM) synthesized on the basis of the 2,5-AM ring combines well with GLUT5 in Chinese hamster ovary (CHO) cells and can be used as a fluorescent probe targeting GLUT5 [ 162, 163]. However, it should be noted that the accumulation of NBDM probes in cells is limited, resulting in inadequate fluorescence reporting. Recently, a novel fluorescent glycoconjugate was reported as a GLUT5 probe [164]. This fluorescent glycoconjugate is constructed with 2,5-AM as the fixed fructofuranose ring and various coumarins (Cou) as the fluorescent fraction (Man-Cou probe), which can target GLUT5 in malignant cells for viable cell metabolic analysis, and the positive response does not appear in normal cells. Compared to previously developed probes, the improved Man-Cou probe can process samples in only 10 minutes, which can be used for rapid on-site high-throughput diagnosis [165]. 

Although many glycoconjugates have been synthesized for cancer research, diagnosis and treatment, GLUT5-mediated uptake is often limited by many factors that produce uncontrollable losses. It must be emphasized that the molecular structural size and hydrophilicity of the conjugate are important factors affecting the efficiency of GLUT5-mediated drug delivery, and these two factors should be prioritized in the synthetic design of novel bioactive or imaging agents [166]. 

## New Directions and Methods for GLUT5 Research in the Future

The development of new biochemical research tools is undoubtedly an effective way to accelerate the discovery of the structure and function of GLUT5 and its contribution to the pathogenesis of disease. As shown in Figure 2, GLUT5 proteins are also modified by glycosylation. Glycosylation is a kind of posttranslational modification (PTM) that is common in eukaryotic cells and involves the addition of glycan molecules to amino acid residues of polypeptide chains. A large number of studies have confirmed that glycosylation modification plays an important role in the evolution of cancer and other diseases [ 167, 168], but unfortunately, the role played by glycosylation on the GLUT5 protein has not been adequately investigated. 

Labeling target proteins with gene-encoded fluorescent proteins ( *e.g*., green fluorescent protein) is a common strategy for studying protein function in living cells. However, certain types of bioactive molecules, such as glycans, and some biological reaction processes, such as PTM, cannot be observed by fluorescent protein labeling [169]. The biological activities of organic molecules linked by carbon-carbon bonds ( *e.g*., sugars) within living cells are an important way for us to understand changes in cellular physiology or pathology, but it is difficult to observe these biological activities in the native environment. The ability of selective chemical reactions to orthogonalize to multiple functions in biological systems is an important tool in the field of chemical biology. The most representative example is the use of azides. Azides are unique biocoupling chemical agents, and their Staudinger ligation with phosphines and the [ 3+ 2] cycloaddition reaction with alkynes (copper(I)-catalyzed azide-alkyne cycloaddition, CuAAC) (called “click chemistry”) catalyzed by Cu ^+^ are widely used in chemical biology research [ 170 , 171]. Staudinger ligation is biocompatible and can be performed in living animals, but there are disadvantages in that phosphines are susceptible to air oxidation and difficult to synthesize; click chemistry does not require phosphines, but the catalyst Cu ^+^ that must be present is significantly toxic to both bacteria and mammalian cells, which limits the application of click chemistry in living cells [172]. 

**Figure 7:**
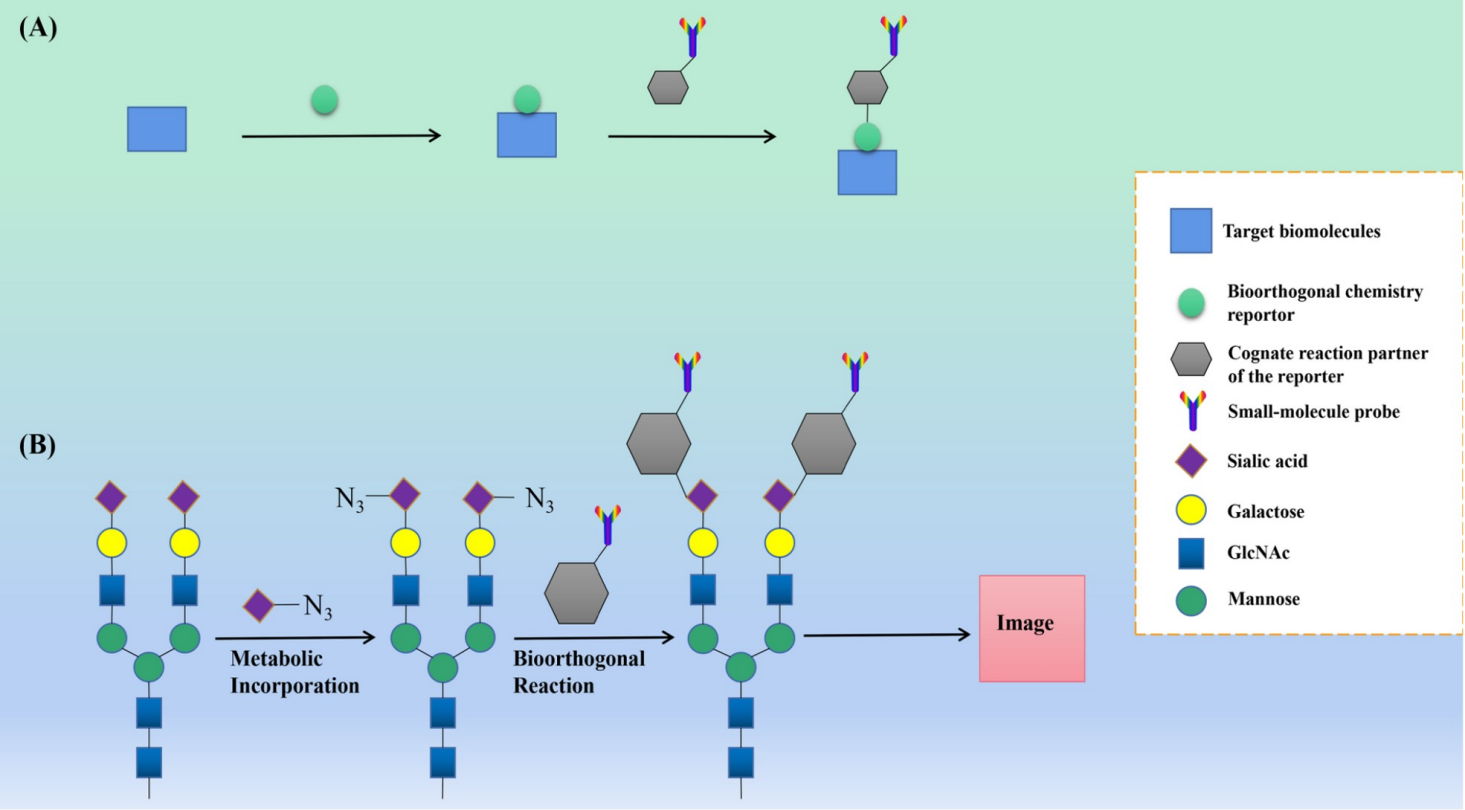
Figure 7 Bioorthogonal chemistry (A) Schematic representation of bioorthogonal chemistry approach for labeling of a targeted biomolecule with a small-molecule probe [176, 177]. (B) Schematic illustration of fluorescence imaging of sialoglycans in living animals by bioorthogonal chemistry [181 ,182].

To track the activity of glycans in living cells, Bertozzi and his colleagues [173] improved click chemistry and pioneered the development of ring strain-activated [3+2] alkyne-azide cycloaddition reactions. Specifically, the reaction of cyclooctyne, which has high tension and electron-absorbing groups, with azide greatly increases the reaction rate without any catalyst. This modified click chemistry overcomes the cytotoxicity of Cu ^+^ in the CuAAC reaction and can be carried out in living cells and even in living animals, thus introducing the concept of “bioorthogonal chemistry”, *i* . *e*., ″click chemistry in living organisms″ [ 174– 176]. Performing bioorthogonal chemistry involves two sequential steps ( Figure 7A): (1) incorporation of the bioorthogonal reporter into the target biomolecule and (2) bioorthogonal reaction between the bioorthogonal reporters and their homologues attached to external chemical probes. A significant advantage of bioorthogonal chemistry is its applicability to all biomolecules, including lipids, proteins, glycans, and nucleic acids [177]. Moreover, most of the reagents involved in bioorthogonal chemistry can be degraded *in vivo*, which further supports the safety of performing bioorthogonal chemistry *in vivo* [178]. For their outstanding contributions to the field of bioorthogonal chemistry, Bertozzi shares the 2022 Nobel Prize in Chemistry with two other pioneers in the field of click chemistry-Sharpless and Meldal [179].

The emergence of bioorthogonal chemistry based on click chemistry has greatly promoted the development of the fields of biochemistry and pharmacy. Currently, a variety of newly developed bioorthogonal chemistries have been applied to identify and characterize proteoglycan modifications on the cell surface ( Figure 7B), and this class of techniques is emerging in cancer diagnosis and targeted therapy [ 180– 182]. More importantly, the affinity between nanoparticles modified by click chemistry compounds and azide-labelled cancer cells is significantly enhanced, which greatly improves the drug delivery capability of nanoparticles, and the combination of bioorthogonal chemistry-nanoparticle technology shows great advantages and potential in the field of nanomedicine [ 183– 184]. Zhou *et al* . [185] preliminarily verified the feasibility of delivering compounds chemically synthesized by click into cancer cells via GLUT5 in MCF-7 cells. In addition, the fluorinated Man-Cou analogue (ManCou-F) synthesized by click chemistry modification has been proven to be a good PET imaging probe, which exhibited GLUT5 preference properties and could clearly show a high level of GLUT5 expression in MCF-7 cells without cytotoxicity [186]. 

**Figure 8:**
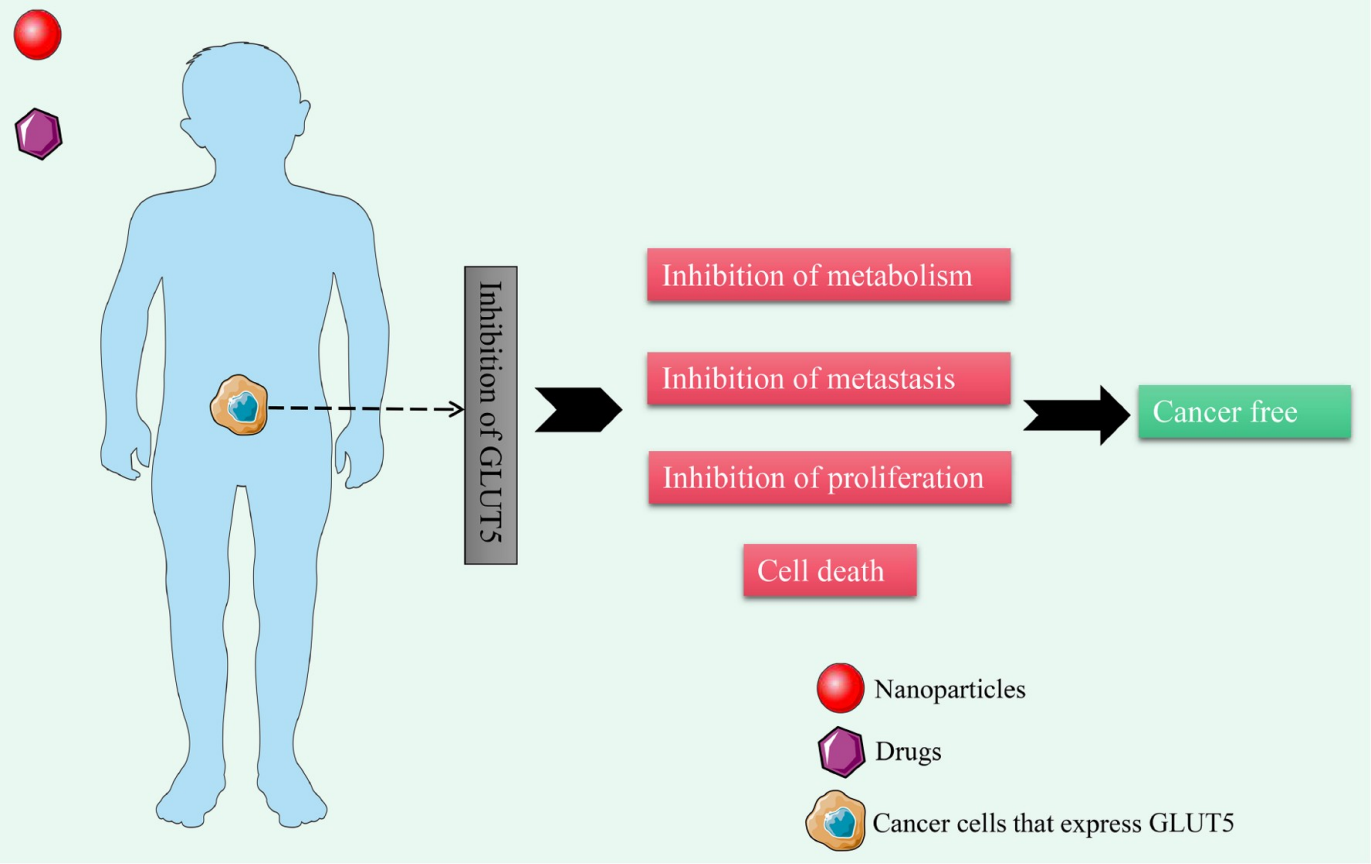
Figure 8 Targeting GLUT5 on the surface of cancer cells by nanoparticles carrying specific drug molecules may be an effective strategy for treating certain cancers in the future [ 183 , 184 ]

Perhaps, in the future, the application of bioorthogonal chemistry can not only analyze the structure and function of GLUT5 but also target GLUT5 on the surface of cancer cells or deliver specific drug molecules by combining with nanoparticle technology ( Figure 8).

## Conclusion and Perspectives

**Figure 9:**
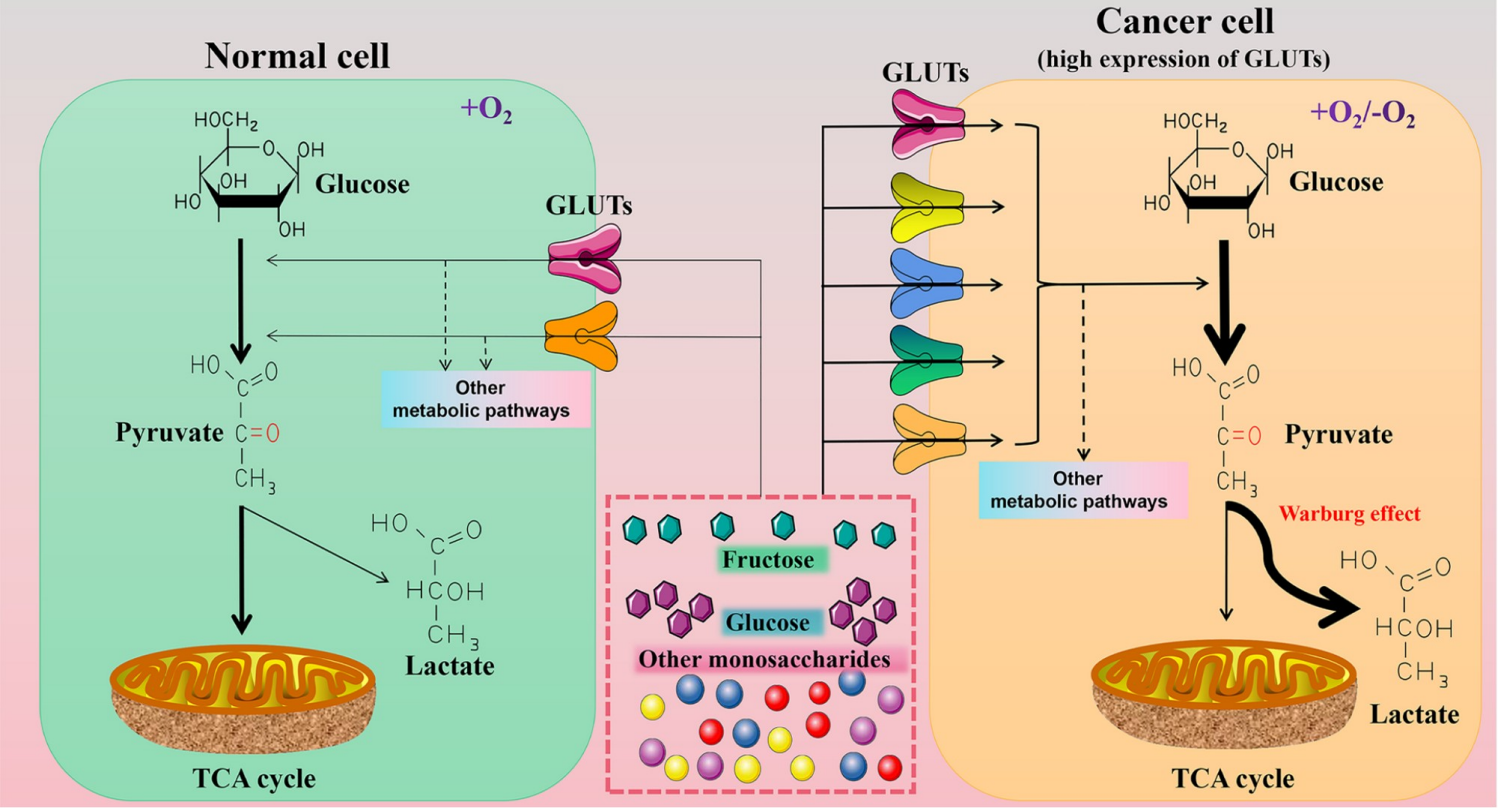
Figure 9 High levels of GLUTs promote cancer cell metabolism On the left of the illustration, cells expressing normal levels of GLUTs are mainly used for TCA cycle under aerobic conditions. On the right side of the illustration, cancer cells that express high levels of GLUTs have high uptake of extracellular monosaccharides, and the products of the cancer cell glycolytic pathway are primarily used for lactate production in both aerobic and hypoxia conditions (Warburg effect) [188 ,189,191].

Metabolic disorders of energy substances in the body are one of the causes of many human diseases. Today, cancer has become one of the leading causes of human death. Although the mechanism of cancer development has been studied in the past hundred years, the changes in metabolic pathways and regulatory mechanisms in cancer cells remain to be elucidated in depth. Metabolic dysregulation of cells is considered to be one of the hallmarks of carcinogenesis and often drives or exacerbates cancer progression, as altered metabolic status in cancer cells is often attributed to dysfunction of certain oncogenes or cancer suppressor genes [187]. As Otto Warburg proposed 100 years ago, tumor cells prefer to use glucose for glycolysis to produce lactate rather than undergo the TCA cycle, even in aerobic conditions [188]. However, since glycolysis produces significantly less ATP per molecule of glucose than the TCA cycle, tumor cells must take in more glucose to meet their own needs [189]. Rapid growth and proliferation force cancer cells to face tremendous nutritional stress, which can be temporarily relieved by massive uptake of glucose from the external environment or the breakdown of lipids stored in intracellular lipid droplets [190]. However, after a large amount of glucose and lipid consumption, cancer cells have to seek new sources of energy to meet their enormous demands, and at this point, fructose acts well as an alternative energy and carbon source. This implies that GLUTs, which transport glucose and fructose, play an important role in the metabolic changes of tumor cells ( Figure 9). In addition, GLUTs, which are widely distributed on the cell surface, are often the “first line” stimulated by external oncogenic stimuli. Numerous previous studies have demonstrated that almost all GLUTs, including GLUT5, are abnormally expressed in different types of cancer [191]. Therefore, targeted inhibition of GLUT expression provides a potential new strategy for the treatment of cancer.

Fructose can induce the upregulation of GLUT5 expression in CRC cells cultured under hypoxia, thereby enhancing the capacity of malignant cells to adapt to hypoxia and improving survival [118]. This suggests that fructose-GLUT5 may play a key role in meeting the minimum environmental requirements for malignant cell survival. GLUT5 plays an important role not only in the digestive system but also in other systemic diseases [192]. For example, downregulating GLUT5 expression in the intestine of diabetic model rats can significantly enhance the efficacy of hypoglycemic drugs [193]. Therefore, regulating GLUT5 expression or targeting GLUT5 to regulate cellular metabolism is a very promising potential target for the treatment of diseases such as cancer. For this, several questions need to be further addressed in future studies related to GLUT5: (1) What is the role of GLUT5 in the formation of the niche before liver metastasis from intestinal tumors? (2) Is GLUT5 related to tumor metastasis organ tropism? (3) What are the functions of GLUT5 that are essential for glycosylation modifications on GLUT5 protein? (4) Can the gut microbiota actively affect the expression level of GLUT5 in intestinal cells? (5) Can changes in tissue GLUT5 expression be a clinical diagnostic criterion for certain diseases? (6) What are the sites of the amino acid residues that can determine the fructose-sensing ability of GLUT5? (7) In addition to X-ray diffraction, cryo-electron microscopy and other techniques, what are the new techniques that can be developed to further determine the multiple structural states of GLUT5 during one transport cycle? 

In fact, in addition to fructose, GLUT5 can also transport L-sorbose, the C-3 epimer of D-fructose, into cells. This is a rare sugar that induces apoptosis after entering cancer cells through GLUT5 [194]. This suggests that the contribution of GLUT5 to normal or cancer cells may be more than related to fructose. Currently, enhanced intestinal GLUT5 expression has been found in obese, overweight and individuals with type 2 diabetes, which provides strong clinical evidence for targeting GLUT5 to treat metabolic diseases [ 195– 197]. Therefore, it is necessary to carry out more adequate and further structural and mechanistic studies of GLUT5 and design rational GLUT5 drug delivery systems. In this way, not only metabolic diseases but also certain metabolism-related/induced cancers can be treated more specifically, and the development of more targeted GLUT5 therapeutic drugs with strong targeting and significant effects is also worth looking forward to. 
